# Cellular and Molecular Dynamics of Th17 Differentiation and its Developmental Plasticity in the Intestinal Immune Response

**DOI:** 10.3389/fimmu.2017.00254

**Published:** 2017-03-31

**Authors:** Suniti Bhaumik, Rajatava Basu

**Affiliations:** ^1^Division of Anatomic Pathology, Department of Pathology, University of Alabama at Birmingham (UAB), Birmingham, AL, USA; ^2^Division of Molecular and Cellular Pathology, Department of Pathology, University of Alabama at Birmingham (UAB), Birmingham, AL, USA

**Keywords:** Th17, immune response, developmental plasticity, intestine, T helper cells

## Abstract

After emerging from the thymus, naive CD4 T cells circulate through secondary lymphoid tissues, including gut-associated lymphoid tissue of the intestine. The activation of naïve CD4 T cells by antigen-presenting cells offering cognate antigen initiate differentiation programs that lead to the development of highly specialized T helper (Th) cell lineages. Although initially believed that developmental programing of effector T cells such as T helper 1 (Th1) or T helper 2 (Th2) resulted in irreversible commitment to a fixed fate, subsequent studies have demonstrated greater flexibility, or plasticity, in effector T cell stability than originally conceived. This is particularly so for the Th17 subset, differentiation of which is a highly dynamic process with overlapping developmental axes with inducible regulatory T (iTreg), T helper 22 (Th22), and Th1 cells. Accordingly, intermediary stages of Th17 cells are found in various tissues, which co-express lineage-specific transcription factor(s) or cytokine(s) of developmentally related CD4 T cell subsets. A highly specialized tissue like that of the intestine, which harbors the largest immune compartment of the body, adds several layers of complexity to the intricate process of Th differentiation. Due to constant exposure to millions of commensal microbes and periodic exposure to pathogens, the intestinal mucosa maintains a delicate balance between regulatory and effector T cells. It is becoming increasingly clear that equilibrium between tolerogenic and inflammatory axes is maintained in the intestine by shuttling the flexible genetic programming of a developing CD4 T cell along the developmental axis of iTreg, Th17, Th22, and Th1 subsets. Currently, Th17 plasticity remains an unresolved concern in the field of clinical research as targeting Th17 cells to cure immune-mediated disease might also target its related subsets. In this review, we discuss the expanding sphere of Th17 plasticity through its shared developmental axes with related cellular subsets such as Th22, Th1, and iTreg in the context of intestinal inflammation and also examine the molecular and epigenetic features of Th17 cells that mediate these overlapping developmental programs.

## Introduction

When an antigen-inexperienced CD4 T cell encounters its cognate antigen in the secondary peripheral lymphoid tissues, it differentiates into T effector cells guided by a microenvironment consisting of a diversity of antigens, antigen-presenting cells (APCs), and other innate immune cells. Amidst the complex environment, lineage-specific fate decision of a naïve CD4 T cell toward differentiating into a specialized T helper (Th) cell is contingent on several variable factors: (a) type of pathogen-associated molecular patterns (PAMPs), (b) types of APC, (c) strength of T cell receptor (TCR) stimulation, (d) strength of costimulation, (e) cytokine gradients, (f) nature of cytokine-induced signal transducer and activator of transcription factor (STAT) signaling, (g) induction of lineage-specific transcription factors (TFs), and (h) induction of lineage-associated TFs. Contingent on nature of these variable factors, naive CD4 T cell can be programmed to T helper 1 (Th1) cells producing IFNγ; T helper 2 (Th2) cells producing IL-4, IL-5, and IL-13; Th17 cells producing IL-17A/IL-17F; T helper 22 (Th22) cells producing IL-22, or inducible regulatory T (iTreg) cells producing IL-10 (1, 2). Not only these subsets are characterized by the signature cytokine(s) they produce, each subset is regulated by the induction of a distinct “lineage-specific” or “master” TF. T-box protein expressed in T cells (T-bet), GATA binding protein 3 (Gata-3), aryl hydrocarbon receptor (AhR), retinoic acid-related orphan receptor gamma t (RORγt), and Forkhead box P3 (FoxP3) are lineage-specific TFs of Th1, Th2, Th22, Th17, and iTreg cells, respectively (3–8). Before the induction of the lineage-specific TF, members of the STAT protein family transmit cytokine-mediated signals and kick-starts the initiation of Th differentiation. Out of the seven STATs identified in mouse, STAT1/STAT4, STAT6, STAT3, and STAT5a/b play non-redundant functions in differentiation of Th1, Th2, Th17, and iTreg cells, respectively (9–16). Once each lineage is “fixed” into a committed phenotype, they are expected to grow clonally in a deterministic way without any change of lineage fate.

Despite its relevance, the standard “2-factor” model of Th lineage differentiation, consisting of STAT and master TF-driven differentiation, underscores the multifactorial complexity of Th17 differentiation. Besides the requirement of RORγt, the lineage-specific TF for Th17 differentiation, multiple lineage-associated TFs also play critical roles in regulating Th17 differentiation. CD4 T cell deficient in several TFs such as RORα, AhR, IRF4 (Interferon regulatory factor 4), and BATF (Basic Leucine Zipper ATF-like TF) also show attenuated Th17 differentiation that cannot be restored by overexpression of RORγt (16–22). More recently, IRF4 and BATF have been designated as “pioneer” TFs that act downstream of TCR signaling and bind to the promoters of *Il17a/Il17f* genes for regulating their chromatin accessibility to lineage-specific TFs at the region (23). Therefore, the growing layers of complexity overwhelms the linear narrative of Th17 differentiation as we now appreciate the inherent phenotypic instability or “plasticity” of the Th17 subset that is evident from presence of intermediate phenotypes in various organs, including the intestine.

In the intestine, CD4 T cell differentiation is a highly intricate process. Retinoic acid (RA), a vitamin A metabolite produced by intestinal APCs, is a principal co-factor that promotes iTreg development and inhibits Th17 development (24, 25). Even in presence of IL-6 and TGFβ, RA strongly counteracts Th17 developmental program by reciprocally favoring iTreg development (15, 25, 26). However, despite the robust production of RA by intestinal APCs, the greatest number of Th17 cells develops in the intestine under inflammatory conditions (27). Therefore, it is perplexing how CD4 T cells undergo vigorous Th17 differentiation in a microenvironment that is replete with Th17-counteracting mediators that support iTreg development. Interestingly, a substantial percentage of Th17 cells in the intestinal lamina propria express FoxP3 at some point during their development indicating a dynamic relationship between the iTreg and Th17 cells (28).

**Figure 1:**
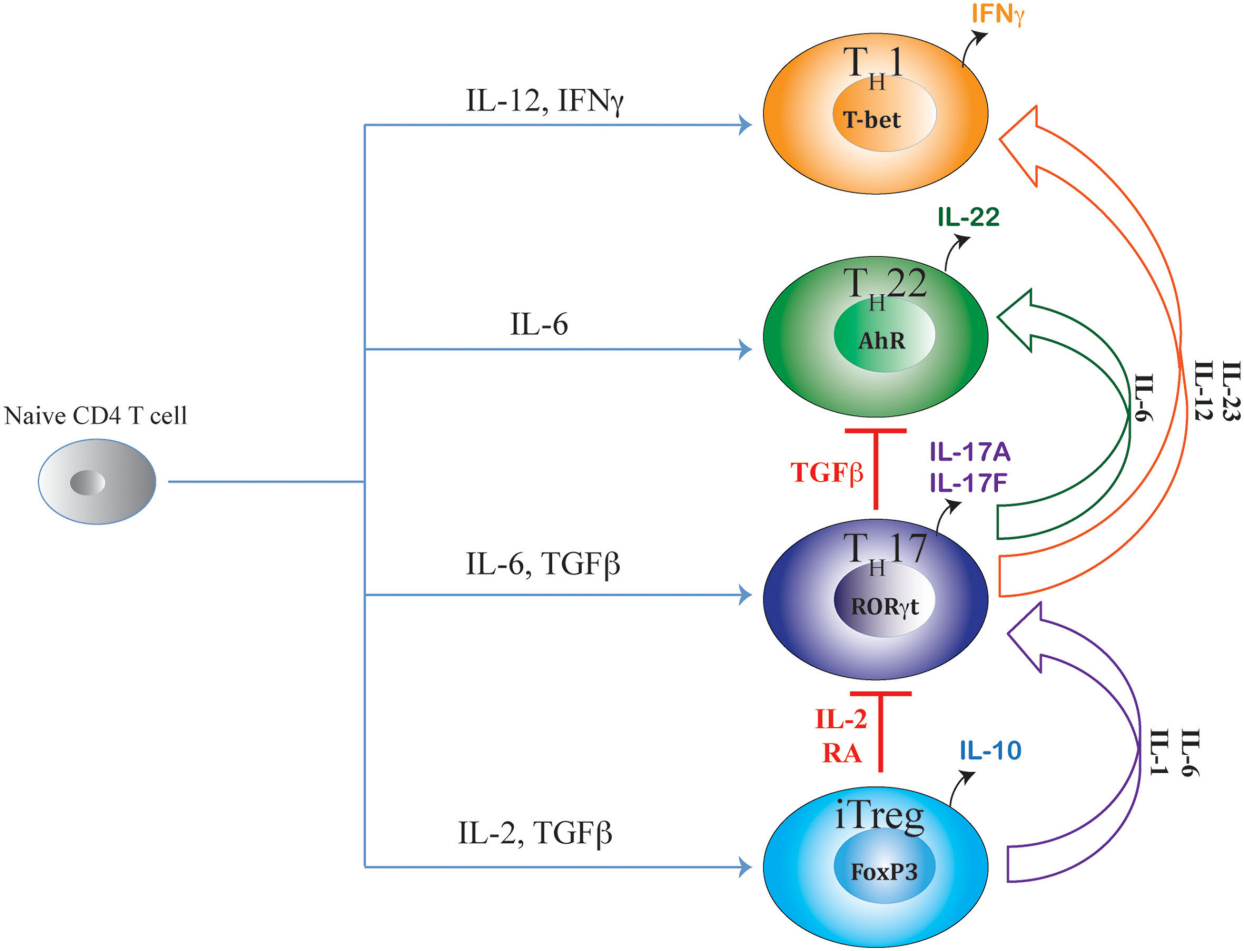
**Shared axes of Th17 differentiation**. Developmental axis of Th17 subset substantially overlaps with developmental axes of iTreg, Th22, and Th1 subsets of T helper cells. While the origin of Th17 differentiation is intrinsically linked with iTreg cells due to their common requirement of TGFβ signaling, Th17 differentiation is also linked with Th22 subset due to the shared requirement of IL-6 signaling. Although proximal signaling events guiding Th17 differentiation are distinct from the Th1 subset, late developmental axis of Th17 is overlapping with Th1 cells as chronic TCR stimulation or action of IL-23 or IL-12 readily converts mature Th17 cells to IFNγ-producing “Th1-like” cells. Accordingly, along the entire developmental axis of Th17 and its related subsets, intermediate phenotypes co-producing FoxP3/IL-17, IL-17/IFNγ, and IL-17/IL-22 are found *in vivo* that can perform beneficial or pathogenic functions depending on the nature of the disease. While IL-2 and retinoic acid (RA) are negative regulators of iTreg–Th17 axis that oppose Th17 differentiation while promoting iTreg differentiation, TGFβ negatively regulates the Th17–Th22 axis as well as the Th17–Th1 axis by suppressing Th22 and Th1 cellular differentiations while facilitating iTreg and Th17 differentiations.

Like Th17 and iTreg cells, Th22 cells, which secrete IL-22 without IL-17 coproduction, are also found in the intestine during inflammation (8). Similar to iTreg cells that share TGFβ signaling with Th17 cells, Th22 cells share a developmental pathway with Th17 cells due to their common developmental requirement for IL-6 (Figure 1). Although Th17 cells were initially believed to be the primary source of IL-22, clear functional differences between Th17 and Th22 cells are evident, as transferred Th22 cells, but not Th17 cells, are able to rescue susceptible mice from enteropathogenic bacterial infection (8). It is intriguing how Th17 and Th22 cells co-evolve in the intestinal environment that is rich in TGFβ—a cytokine that also negatively regulates Th22 differentiation. Another prominent Th subset, which has developmental ties with the Th17 pathway, is the Th1 subset. Unlike Th22 and iTreg cells, proximal signaling events guiding “classical” Th1 differentiation are distinct from Th17 cells. Yet, differentiated Th17 cells frequently transit to “Th1-like” populations under inflammatory conditions of the intestine (29–31). During autoimmune colitis, transferred Th17 population rapidly transit to T-bet-expressing Th1-like Th17 cells leading to aggravated autoimmune response (31). These Th17-derived, Th1-like cells are recognized as a principle pathogenic effector population in several autoimmune diseases, including inflammatory bowel disease (IBD). Although several factors that contribute to the late developmental transition of Th17 precursors to Th1-like cells have been identified, details of how the late developmental axis of Th17 cells overlaps with Th1 cells despite apparent developmental dissimilarities between these two subsets remain to be defined. Due to this intrinsic developmental link of Th17 cells with iTreg, Th22, and Th1 cells, a complex dynamic interaction takes place among different cytokine-induced TFs, lineage-specific TFs, and lineage-associated TFs during Th17 differentiation that strongly influences the fate commitment and plasticity of Th17 cells. This indicates a complex, multifactorial decision-making process during Th17 lineage commitment, warranting detailed study of the developmental relationship with related subsets, which will be discussed in this review.

## “Non-Cytokine” Factors Influencing Th17 Fate Commitment

**Figure 2:**
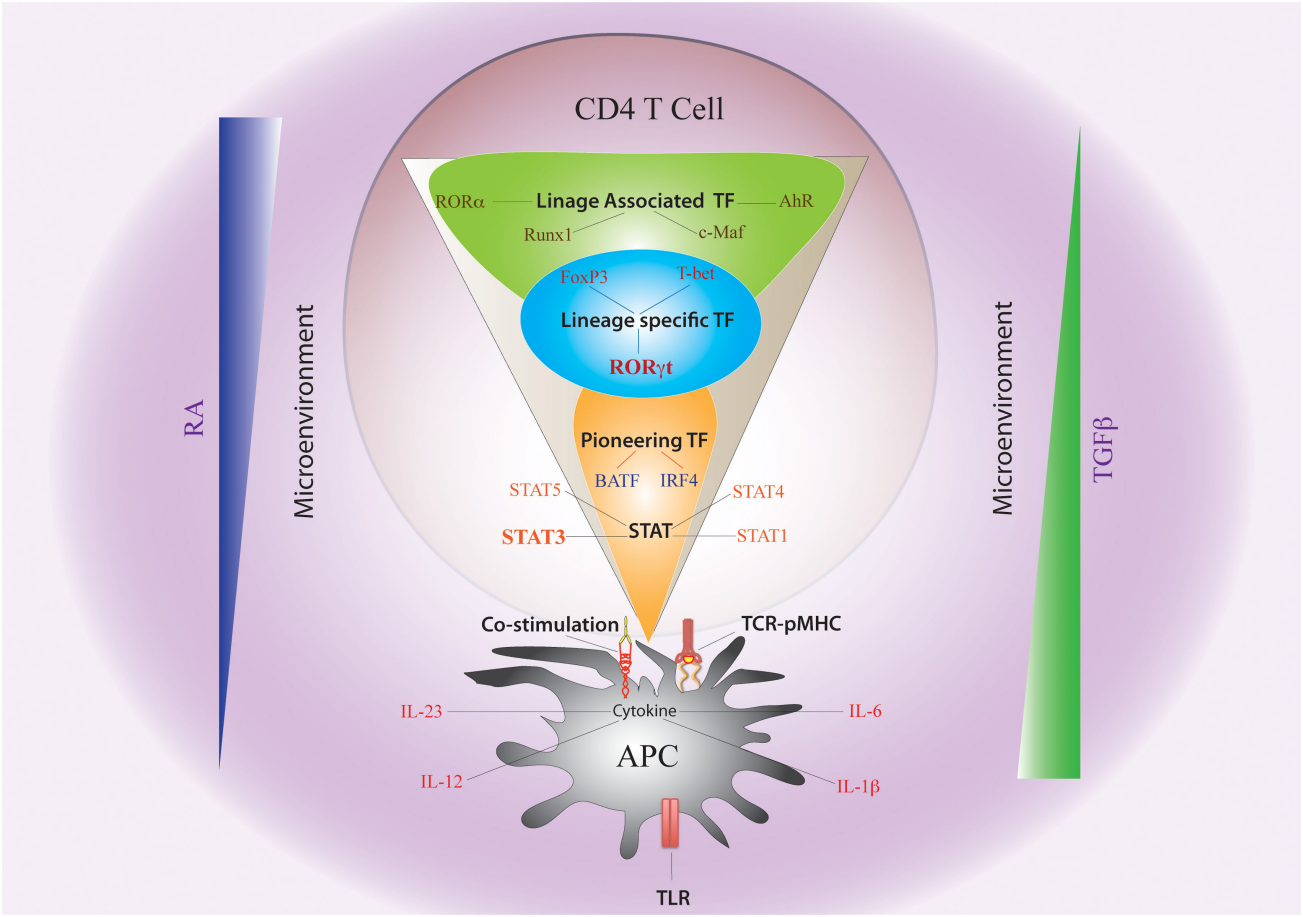
**Non-cytokine Factors and Cytokine-Induced Transcription Factors Governing Th17 Plasticity**. The fate of Th17 differentiation during the journey of a naive CD4 T cell towards becoming an antigen-specific Th17 cell can be broadly dictated by “non-cytokine” factors and “cytokine-induced” transcription factors (TFs). Among “non-cytokine” factors, strength of TCR–pMHC engagement, strength of co-stimulation, nature of pathogen-associated molecular pattern or PAMP–TLR interaction, and nature of APC impact plasticity of Th17 cells. Moreover, the microenvironment of APC–T cell interaction also guides Th17 lineage commitment. An environment rich in retinoic acid (RA) or TGFβ, which prevails in the intestine, also influences Th17 plasticity. The entire landscape of Th17 programing that is initiated by “cytokine-induced” TFs can be divided into three sequential terrains (orange, blue, and green). During its journey through the first terrain, TCR signaling coupled with co-stimulation and other “non-cytokine” factors cooperate with APC-generated cytokines to induce STAT proteins (STAT3, STAT1, STAT4, and STAT5) and “pioneer” TFs like BATF and IRF4 (Orange). The STAT proteins and the pioneer TFs then jointly initiate the lineage-specific developmental programing by inducing STAT-responsive and IRF4/BATF-responsive genes, which include activation of master TF (RORγt) of Th17 cells. During its transition through the second terrain, STAT protein-induced RORγt jointly cooperates with the “pioneer” TFs to alter chromatin accessibility of key Th17-specific genes by epigenetic modification (Blue). During this point of time, the lineage-associated TFs are also induced during Th17 differentiation by both “non-cytokine” and “cytokine-mediated” signaling. In the final terrain of Th17 programming, an orchestration of complex networking of signaling events modulated by the lineage-specific TF (RORγt) in association with lineage-associated TFs (e.g., c-Maf, AhR, Runx1, etc.) determine the stability of the Th17 developmental program through integration of various additional pro-inflammatory and anti-inflammatory environmental cues. Although STAT3-induced RORγt predominantly drives Th17 programing, other STAT proteins (like STAT1, STAT5) and TFs (like T-bet and FoxP3) that alters Th17 lineage stability are also induced contingent on the initial priming environment. Lineage commitment or plasticity of Th17 cells is the outcome of the interplay of these variable factors acting across all the three levels.

When a naïve CD4 T cell engages with APC *via* TCR–pMHC complex, the fate of Th differentiation is broadly dictated by “non-cytokine” factors as well as by “cytokine-induced” TFs (Figure 2). The complex system of molecular communications from non-cytokine factors initially predisposes the naïve CD4 T cells to a specific differentiation pathway prior to the action of cytokine. The non-cytokine factors that influence Th17 differentiation are as follows: (a) nature of microbial antigen or PAMP, (b) nature of APC, (c) strength of TCR–pMHC interaction, and (d) strength of costimulation.

### PAMP and APC

Pathogen-associated molecular patterns (PAMPs) present on the cell wall or cell membrane of microbes stimulate APC in different ways to induce a wide variety of cytokines. Human monocytes pulsed with the fungus *Candida albicans* and the bacteria *Staphylococcus aureus* produce IL-6, TNFα, and IL-23, whereas IL-12 is induced exclusively by the fungus but not by the bacteria (32). Therefore, fungal antigens, not bacterial antigens, specifically stimulate APCs to produce IL-12, resulting in the conversion of naïve CD4 T cells into fungus-specific Th17 cells that produce both IL-17 and IFNγ. Moreover, β-glucan, a major PAMP isolated from fungal cell wall, induces production of an inflammatory lipid mediator, Prostaglandin E2 (PGE2), which plays a pivotal role in Th17 differentiation (33). Inhibition of PGE2 synthesis drastically reduces IL-23 production by β-glucan-activated APCs, suggesting that endogenous PGE2 amplifies IL-23 synthesis in response to the *C. albicans*-associated PAMP. Besides the specificity of PAMPs on microbes, the specificity of the T cell activating stimulus also depends on the type of APC recruited to the site of interaction. For example, intestinal CD103^+^ DCs are much more efficient than CD103^−^ DCs in TGFβ-mediated conversion of naive CD4 T cells into iTreg cells. This is due to their specialized capacity to produce higher levels of retinaldehyde dehydrogenase (RALDH2), which enables them to convert retinal, a Vitamin A derivative, into RA (24, 25). Accordingly, intestinal CD103^+^ DCs, not splenic DCs, inhibit Th17 differentiation and facilitate iTreg development by overcoming the Th17-promoting effect of IL-6 (25). Moreover, some APCs can also preferentially induce specific cytokines that block Th17 differentiation. Type 1 IFN-secreting plasmacytoid DCs inhibit *Bordetella pertussis*-specific Th17 differentiation leading to exacerbation of respiratory disease (34). As TLR7/TLR9 is preferentially expressed in plasmacytoid DCs, it is likely that TLR7/TLR9-activating PAMPs on the pathogen promote IFNα secretion from plasmacytoid DCs, which inhibits Th17 differentiation by the suppressive effects of STAT1 signaling induced by Type I IFNs (35–37).

### Strength of TCR–pMHC Interaction

Strength of TCR stimulation is another independent variable influencing Th lineage commitment. Peptide/MHC complexes that interact more strongly with TCR favor generation of Th1-like cells, while those that bind weakly favor Th2-like cells as low concentration of peptide increases GATA3 expression to facilitate Th2 differentiation (38). Compared to wild-type peptide, mutant human collagenase IV peptides with weaker affinity for their cognate TCR also elicit an IL-4-producing Th2 response while mutant peptides with higher affinity prime an IFNγ-producing Th1 response (39). Weaker affinity-driven Th2 response might be advantageous to an organism, as autoreactive T cell clones emerging from thymic selection at the lower end of affinity spectrum will either be anergic or release Th2 cytokine, thereby limiting the extent of inflammatory damage. However, conflicting findings exist regarding the role of TCR strength on Th17 differentiation. In mouse model, decreased strength of TCR stimulation preferentially reduced IL-17A expression in a calcineurin/NFAT-dependent manner (40). Moreover, despite being exposed to Th17-promoting cytokines, naïve CD4 T cells receiving weak TCR signals preferentially express FoxP3 and resist differentiation to Th17 pathway (41, 42). Accordingly, deficiency of Tec family tyrosine kinase Itk, which is activated upon TCR signaling, results in impairment of Th17 differentiation with their concomitant divergence to FoxP3-inducible iTreg cells (43). However, Itk deficiency in CD4 T cells, besides defective NFATc1 sssdifferentiation under low avidity TCR stimulation (44). This raises the possibility that downstream signaling pathways contingent on TCR signaling strength can predispose differentiation of a naïve CD4 T cell along iTreg/Th17/Th1 axis.

In apparent contrast, low-strength stimulation of CD4 T cells has also been shown to favor a Th17 response that is explained by a failure of high-strength TCR-activated Th17 cells to induce binding of NFATc1 to the IL-17 promoter (45). NFATc1 has been reported to be a crucial TF for regulating IL-17 promoter activity in response to TCR signaling.

Interestingly, high induction of IFNγ also takes place along with IL-17 in low-strength TCR-stimulated Th17 cells, suggesting that TCR strength plays a role in plasticity of Th17 cells. Repetitive TCR stimulation can also facilitate transition of Th17 cells to Th1 cells (29). Hence, both along the early iTreg–Th17 and late Th17–Th1 developmental axes, TCR signal strength is one of the contributory factors in determining Th17 plasticity. In conclusion, both affinity and dose of the antigen *via* strength of TCR stimulation are instrumental to bias initial programing of differentiation of a developing antigen-experienced CD4 T cells toward a particular Th lineage.

### Strength of Costimulation

Besides TCR signal, differentiation of naive T lymphocytes into effector cells requires additional signals provided by costimulatory pathways mediated by B7:CD28 and CD40:CD40 ligand (CD40L) interactions. CD28 costimulation plays a critical role along the iTreg–Th17 axis of differentiation. Lack of CD28 costimulation impairs the ability of naïve T cells to differentiate into iTreg cells in the periphery in an IL-2-dependent manner (46, 47). Conversely, high CD28 costimulation inhibits Th17 development indicating that strength of CD28-B7 engagement can reciprocally modulate the differentiation of naïve CD4 T cells along the iTreg–Th17 developmental axis (48). As strong costimulation amplifies IL-2 induction that is known to inhibit Th17 differentiation, it is likely that strength of CD28 stimulation can negatively influence Th17 differentiation while facilitating iTreg differentiation *via* an IL-2/STAT5-dependent pathway. Besides CD28 costimulation, CD40–CD40L crosstalk is required for optimal Th17 differentiation. Although IL-12 production from DC is one of the mechanisms by which CD40-mediated signaling exerts its influence on priming of effector T cells, the requirement of CD40–CD40L interaction between APCs and T cells also involves other mechanisms such as production of other cytokines from DCs in a contextual manner (49, 50). During Th17 differentiation, a complex interaction between strong antigenic signals and PAMP-dependent pathogenic stimuli induce CD40L expression, which increases IL-6 production from DCs for facilitating Th17 differentiation (41). Upon T cell priming by zymosan-exposed *Cd40^−/−^* DCs, Th17 expansion is partially rescued by addition of exogenous IL-6. Accordingly, immunization of *Cd40^−/−^* mice with a high antigen dose results in an impaired Th17, but not Th1 differentiation. Therefore, the strength of costimulation along with strength of TCR engagement and nature of PAMP can predispose an antigen-experienced CD4 T cells toward a specific lineage.

## Cytokines and Cytokine-Induced Transcription Factors Influencing Th17 Fate Commitment

Decades ago, it was conceptualized that activated T cell clones preferentially producing IFNγ are Th1 subsets that are instrumental in eliminating intracellular pathogens, while those producing IL-4, IL-5, and IL-13 are Th2 subsets that play a vital role in expelling extracellular helminths (51, 52). This selective cytokine production was thought to be stable as clonally propagated Th1 or Th2 clones continued to produce their signature cytokines without any inter-conversion or fate reversal (53). Although the discovery of Th1/Th2 subsets of CD4 Th cells revolutionized our understanding of adaptive immune response, it also fostered a rigid picture of the stability of these cells (53, 54). Currently, the situation we confront is more complex. IFNα, IFNγ, and IL-12 are now considered to be key innate cell-derived modulators of Th1 differentiation, while IL-25, IL-33, and thymic stromal lymphoprotein (TSLP) are key innate immune cell-derived cytokines governing Th2 differentiation (55–58). The CD4 T cell repertoire was substantially expanded with the discovery of iTreg, Th17, and Th22 cells. At present, Th17 differentiation is emerging as a highly complex process involving multiple cytokines and TFs that outnumber other lineages such as Th1 and Th2 subsets. Whereas, IL-6 and TGFβ constitute the primary cytokines initiating Th17 development, IL-21, IL-1β, and IL-23 play distinct roles in guiding the Th17 differentiation process to a committed state. While, RORγt and STAT3 are essential TFs required for Th17 lineage differentiation, other TFs such as AhR, IRF4, BATF1, and Runx1 are also required to regulate optimal Th17 development.

### The Critical Axis of IL-6/IL-21/STAT3/RORγt

IL-6, an acute phase protein, is a critical differentiation factor for the generation of Th17 cells (27, 59). Binding of IL-6 to its co-receptors IL-6R and gp130 results in activation of STAT3, which induces IL-17 *via* activation of RORγt (19, 60). In STAT3-deficient CD4 T cells grown under Th17 polarizing conditions, there is complete abrogation of IL-17 induction. Therefore, both STAT3 and RORγt co-operatively induce optimal expression of IL-17 (19). As overexpression of RORγt alone is sufficient to direct *Il17* transcription in the absence of exogenous cytokine, it is considered as the “master” TF of Th17 cells. However, besides direct transcriptional activation of *Il17a/Il17f* locus, other critical functions of RORγt during Th17 development are poorly understood. Despite the critical importance of STAT3 in Th17 differentiation, its overexpression fails to induce optimal Th17 differentiation in absence of RORγt, suggesting that STAT3 co-operates with RORγt to induce optimal Th17 differentiation (19).

IL-6 also induces the production of IL-21 in Th17 cells, which can, in turn, induce expression of IL-23R, subjecting Th17 cells to the effects of IL-23 (19). Although the generation of Th17 cells is reported to be impaired in the absence of IL-21 signaling, Th17 cells can develop in the absence of IL-21/IL-21R signaling (61–64). It is likely that IL-21 influences pathogenicity of Th17 cells in autoimmune diseases by enhancing the effect of IL-23 on Th17 cells, which promotes Th1 competence of Th17 cells. Curiously, an IL-6-independent pathway of Th17 development has also been demonstrated specifically in tissues where RA is absent. In *Il6^−/−^* mice, Th17 cells were detected in the peripheral secondary lymphoid organs but not in the intestinal lamina propria where RA is produced at a higher concentration (65). It seems that due to copious production of RA by resident CD103^+^ DCs in the intestine, IL-6 becomes essential for Th17 development as it overcomes the suppressive effect of RA on Th17 differentiation.

We have recently demonstrated that IL-6 alone is critically required, but not sufficient to overcome RA-mediated suppression of Th17 differentiation (26). Short-lived STAT3-induction by IL-6 inadequately opposes RA-driven STAT5 induction resulting in suppression of Th17 differentiation in presence of IL-6 both *in vitro* and in the intestinal lamina propria during acute intestinal inflammation. STAT3 activation is also a dominant mechanism to suppress FoxP3 expression during Th17 differentiation. The suppressive effect of STAT3 on FoxP3 is independent of RORγt, as STAT3-deficient Treg cells fail to downregulate FoxP3 expression in presence of IL-6, while RORγt-deficient Treg cells are able to downregulate FoxP3 in presence of IL-6 (66). However, studies from our group have shown that only in intestinal tissue where RA has a dominant presence, transient nature of IL-6-induced STAT3 fails to downregulate FoxP3 expression due to strong STAT5-depenedent FoxP3-activating property of RA (26). In other tissues devoid of high levels of RA, STAT3 activation by IL-6 efficiently represses FoxP3 transcription. Therefore, whereas both STAT3 and RORγt are critical for IL-17 expression, STAT3 alone is sufficient for effective suppression of FoxP3. In the context of a tissue environment such as the intestinal mucosa, where both TGFβ and RA are produced in abundance to facilitate iTreg programming, STAT3 signaling might be vital to attenuate FoxP3 expression for favoring Th17 development.

Interestingly, in addition to its STAT3 activating potential, IL-6 also activates STAT1 in CD4 T cells (67–69). Under Th17 differentiation conditions, *Stat3^−/−^* CD4 T cells assume a Th1 phenotype, suggesting that STAT3 is a master regulator that skews Th17 responses away from Th1 pathway (19, 70). Accordingly, activated lamina propria lymphocytes from STAT3-deficient mice generate a robust Th1 response that is several folds higher than WT mice along with a completely abrogated Th17 response (70). It is likely that besides its role in induction of *Il17a/f* transcription, STAT3 also participates in regulating Th17 plasticity as it counter-regulates Th1 programing during Th17 differentiation. STAT3 also drives positive epigenetic modifications of its target genes during Th17 development. STAT3-bound genes such as *Il17a, Il17f, Il21*, and *Il6ra* contain permissive chromatin marks in WT cells, but these marks are absent or reduced in *Stat3*-deficient Th17 cells (71). Although IL-6 does not appear to be required to maintain a stable Th17 phenotype post-differentiation, a role for on-going STAT3 signaling cannot be excluded in regulating plasticity of Th17 lineage (29). It remains a distinct possibility that in addition to directly program CD4 T cells toward Th17 pathway *via* RORγt activation, STAT3 signaling also counteracts STAT1 activation, which is essential to prevent development of Th1 programming during Th17 development. Besides STAT3, RORγt also plays a role in Th17 stability as its overexpression leads to transcriptional repression of T-bet (72). The entire interaction between RORγt and T-bet forms a feedback loop during Th17 differentiation as T-bet, once expressed, also act directly to silence *Rorc* locus (72). Therefore, besides direct transcriptional activation of *Il17* locus, it is likely that RORγt additionally opposes development of Th1 programing and restricts plasticity of Th17 cells.

### The Controversial Role of TGFβ

The concept of Th17 cells as a stable lineage has been brought into question following characterization of heterogeneous populations of IL-17A-producing CD4 T cells exhibiting various degrees of pathogenicity contingent on the initial milieu of differentiation (73, 74). One of the first reports describing IL-17-producing CD4 T cells as a discrete lineage observed that both IFNγ and IL-4 inhibited differentiation of Th17 cells (37). At the time of this discovery, TGFβ was already recognized as a cytokine that potently inhibited Th1 and Th2 cell differentiation by suppressing T-bet and GATA3 TFs, respectively (75). Accordingly, TGFβ was found to be essential for optimal Th17 differentiation due to its combinatorial action of downregulating IFNγ- and IL-4-induced signaling pathways (27). However, two independent reports noted that TGFβ is dispensable for differentiation of human Th17 cells (76, 77). Soon after, the dispensability of TGFβ in human Th17 differentiation was contested and three studies reestablished TGFβ as a critical factor, which works in concert with inflammatory mediators, including IL-1, IL-21, IL-6, and IL-23, for inducing human as well as mouse Th17 differentiation (78–80).

Remarkably, the dispensability of TGFβ in Th17 differentiation resurfaced again when it was shown that there could be two pathways to Th17 differentiation. While one is dependent on TGFβ, which induces differentiation into “non-pathogenic” Th17 cells, the other is independent of TGFβ and induces differentiation into “pathogenic” Th17 cells (74). It was shown that TGFβ-independent “pathogenic” and TGFβ-dependent “non-pathogenic” subsets of Th17 are phenotypically and functionally dissimilar, with more than 2,000 genes differentially expressed between the subsets. While RORγt was induced in both populations, T-bet was selectively upregulated in “pathogenic” Th17 cells, which showed much higher degree of plasticity in transitioning into Th1-like cells. This is likely due to the effects of TGFβ to potently suppress T-bet-driven Th1 development and restrict transition of TGFβ-dependent “non-pathogenic” Th17 population into Th1-like cells. Despite convincing proof of a TGFβ-independent Th17 developmental pathway linked to its pathogenic functionality, a conundrum still remains. The necessity of TGFβ-signaling in promoting Th17 differentiation was established by another study, which found that deletion of the *Tgfb1* gene from activated T cells considerably impacted Th17 differentiation resulting in highly reduced frequency of IL-17-producing cells (81). Moreover, TGFβ is widely expressed by immune cells and normal human subjects have >2 ng/ml TGFβ1 in their plasma (82). Therefore, it is difficult to conceive of a tissue environment completely devoid of its presence. In both IBD and experimental autoimmune encephalomyelitis (EAE), Th17 cells grown in presence of TGFβ are also shown to be pathogenic, which clearly limits the correlation of TGFβ with non-pathogenic function of Th17 cells (29, 59, 83).

Nevertheless, one can imagine an inflammatory milieu where the concentration of TGFβ is kinetically altered during progression of inflammation, thereby creating a temporal concentration gradient of TGFβ. If a reduced concentration of TGFβ supports development of pathogenic inflammatory Th17 cells as a result of significant induction of T-bet, an altered environment facilitating high production of TGFβ might ensure homeostasis by converting the same subset into a non-pathogenic population by suppressing or restricting high T-bet induction. Alternatively, a drop in concentration in TGFβ during course of inflammation might enable transition of a non-pathogenic Th17 cells into a T-bet co-expressing pathogenic subset. Either way, it indicates that inherent plasticity of Th17 cells can play a vital role in governing immune homeostasis. This line of reasoning might prompt one to think that fate commitment of Th17 cells is a dynamic phenomenon progressing through a series of intermediate developmental stages. Interestingly, population of Th17 cells found in the gut of Crohn’s disease (CD) patients are heterogeneous in nature. Subsets of Th17 cells have been isolated from gut mucosa of CD patients that co-produce IFNγ as well as FoxP3 (84, 85). The IL-17 and FoxP3 co-expressing cells not only exhibit shared phenotypic characteristics of Th17 and iTreg cells but also show potent suppressor activity *in vitro* (86). Therefore, one cannot preclude the likelihood of developmental overlap among Th17, Th1, and iTreg cell lineages that links iTreg–Th17 axis with Th17–Th1 axis of differentiation (2). It is tempting to hypothesize that in an inflammatory setting, contingent on immediate availability of TGFβ, Th17 differentiation lies in a dynamic flux between iTreg and Th1 differentiation, where the transitional iTreg/Th17 cells serve in a regulatory capacity while the Th17/Th1 cells are pro-inflammatory in nature. Th17 cells that are early exposed to high levels of TGFβ assume a more committed Th17 phenotype due to suppression of T-bet compared to those developing under low concentration of TGFβ as they are less prone to conversion to IFNγ-co-producing Th17 cells.

### The Accessory Roles of IL-23 and AhR

IL-23 was originally described as a cytokine closely related to IL-12 that induced IFNγ from human memory T cells in a STAT4-dependent manner (87). Subsequently, IL-23 was heralded as a necessary cytokine for Th17 development as it was found that IL-23 has a dual capacity of inducing IL-17 as well as IFNγ (37, 88). Whereas IL-23 alone specifically induced IFNγ from CD4 T cells in presence of neutralizing antibody to IL-4, IL-23 induced modest IL-17 induction from CD4 T cells in presence of neutralizing antibody to both IL-4 and IFNγ (37). Later, it was found out that IL-23 is dispensable for Th17 development both *in vitro* and *in vivo* suggesting that IL-23 plays an accessory role in modulating Th17 effector function (27). Indeed, IL-23 signaling promotes differentiation of pathogenic Th17 cells (73). The initial observation regarding kinship of IL-23 to the Th1-inducing cytokine IL-12 and its ability to induce IFNγ *via* STAT4 was further corroborated by studies examining the effect of IL-23 on plasticity of Th17 cells. Differentiated Th17 cells continuously exposed to IL-23 deviate to a Th1-like phenotype (29). Moreover, CD4 T cells lacking IL-23R show a reduced emergence of IFNγ-and IL-17-co-expressing phenotype and do not trigger colitis (89). Accordingly, the switch from Th17 to Th1-like cells depends on IL-23-driven induction of T-bet, indicating the essential role of IL-23 in mediating transition of Th17 into Th1 cells (30). With the use of IL-17A-eYFP reporter mice, which permanently marks IL-17A-producing cells, it was demonstrated in a fate-mapping study that the Th1 cells present in the lymph nodes and spinal cord of mice with EAE were all ex-IL-17A producers and IL-23-deficient Th17 cells did not become Th1 cells.

While evidence of induction of RORγt by IL-23 is lacking, IL-23 plays a vital role in upregulating AhR (8, 22). AhR, a heterodimeric ligand activated TF, is also known to positively regulate Th17 development (22, 90). Although AhR is not required for initial differentiation of Th17 cells, it promotes their expansion and is essential for their production of IL-22. Activation of AhR by its natural ligand 6-formylindolo[3,2-b]carbazole (FICZ), a tryptophan derivative, increases both IL-17 and IL-22 from cultured Th17 cells. Unlike RORγt, forced expression of AhR into CD4 T cells in the absence of cytokines does not lead to IL-17 or IL-22 expression. This suggests that signaling intermediates induced jointly by IL-6 and TGFβ co-operate with AhR to facilitate Th17 development. Intriguingly, AhR also regulates FoxP3 expression, suggesting its critical role in development of iTreg cells (91, 92). Activation of AhR by administration of a synthetic ligand 2,3,7,8-tetrachlorodibenzo-p-dioxin (TCDD) helps in the expansion of FoxP3-expressing iTreg cells. How the high-affinity AhR ligand FICZ promotes Th17 development while the low-affinity synthetic ligand TCDD promotes iTreg remains unresolved. It has been proposed that higher half-life and lower affinity of TCDD for AhR binding, compared to naturally occurring high-affinity ligand FICZ, accounts for its influence on FoxP3-expressing iTreg development. AhR promotes gene transcription by binding to a consensus dioxin response element (DRE) upstream of AhR-inducible genes. In the *Foxp3* promoter as well as in promoters of various cytokine and cytokine receptor genes, including STATs, several DRE have been identified that are capable of binding AhR directly (91, 93). Therefore, a role for AhR in regulating STAT1 and STAT5 during Th17 and iTreg development has been proposed (90, 94, 95). The precise role of AhR in Th17 differentiation remains unclear but there is a distinct possibility that it might play a role in Th17 plasticity along both iTreg–Th17 and Th17–Th1 axes by influencing STAT5 and STAT1 activation, respectively. Our own studies have shown that despite upregulation of AhR in Th17 cells, its activation does not directly influence IL-17 induction but plays a critical role in induction of IL-22 from Th22 cells (8). It is likely that AhR also supports low levels of IL-22 induction from Th17 cells but its additional role in other effector functions of Th17 cells requires further investigation.

### The Importance of IL-1/IL-1R1 Signaling

Besides IL-6, IL-1/IL-1R1 signaling is considered to be essential for the differentiation and commitment of Th17 cells (76, 77). Although IL-1β alone induces IL-17 and RORγt from naïve human CD4 T cells, IL-6 or IL-23 has a synergistic effect on IL-1β-mediated IL-17 induction. Mice deficient in IL-1R1 fail to induce IL-17 upon antigen challenge (96). IL-1R1-deficient mice are also resistant to EAE that is associated with a decrease in frequency of Th17 cells (96, 97). After induction of EAE in a mixed chimera experiment, MOG-specific IL-17 cells are reduced in CNS in IL-1R1-deficient cellular compartment along with a significant increase in FoxP3-expressing cells (97). SIGIRR, a negative regulator of IL-1 receptor, also suppress Th17 cell expansion and Th17-mediated disease (98). IL-1 is also known to counter the inhibitory effect of IL-2 on RORγt and IL-23R expression during Th17 differentiation (99). It has been proposed that specific PAMPs induce APCs to promote IL-1β-mediated Th17 differentiation. While *C. albicans*, a pathogenic fungus, strongly induces both IL-6 and IL-1β from human monocytes, *S. aureus*, a pathogenic bacterium, induces IL-6 alone but not IL-1β, suggesting distinct signaling pathways are induced based on the nature of PAMPs recognized (32). Accordingly, neutralization of IL-1β completely abrogates *C. albicans*-induced Th17 differentiation with marked reduction in RORγt level, whereas neutralization of IL-1β has a less severe effect on *S. aureus*-specific Th17 development.

A clear mechanism to explain the role of IL-1 signaling in Th17 development has been evasive. Contradictory reports exist on the requirement of IL-1/IL-1R1 signaling pathways on intestinal Th17 differentiation based on studies on MyD88-deficient mice (100, 101). But the studies investigating the role of IL-1β in intestinal Th17 development have been performed under steady-state conditions rather than under inflammatory conditions where IL-1β reaches its highest level. Moreover, it has also been argued that critical role of IL-1β in Th17 development during intestinal homeostasis has been obscured by the *ex vivo* stimulation of Th17 cells that exaggerate intracellular IL-17 expression and provoke early commitment to IL-17 production before full effector status is achieved (30, 101). Therefore, the role of IL-1β in Th17 development during intestinal inflammation, particularly in disease models where Th17 cells are known to be protective, remains largely unknown. Recently, our group has demonstrated a novel role of IL-1β in modulating iTreg–Th17 developmental axis during intestinal inflammation caused by enteropathogenic bacteria (26). We found that by downregulating SOCS3, IL-1β enhances the amplitude and duration of IL-6-driven pSTAT3 induction and alters the pSTAT3/pSTAT5 balance in developing T cells to enhance Th17 cell development at the expense of iTreg cell development. As RA production is dominant in the gut microenvironment, IL-1/IL-1R signaling is critical to override dominant STAT5 signaling that supports a tolerogenic environment of the gut, thereby promoting Th17 differentiation at the expense of iTreg development. Accordingly, in intestinal lymphoid tissues but not in other lymphoid tissues of IL-1R1-deficient mice, there is a skewed balance of FoxP3- and IL-17-expressing CD4 T cells with impaired Th17 differentiation. Therefore, it is likely that IL-1β becomes critically required for optimal differentiation of Th17 cells particularly in environments rich in RA, which otherwise suppresses Th17 development.

### The Emerging Roles of IRF4 and BATF

IRF4, a member of interferon-regulatory factor TF previously known to be associated with GATA3-mediated Th2 differentiation, is required for Th17 cell differentiation (102, 103). Besides Th2 and Th17 differentiation, IRF4 is also essential for the development and function of IL-9-producing Th9 subset as well as development of effector Treg subset (104, 105). IRF4-deficient mice are resistant to EAE with a severely reduced frequency of Th17 cells (21). Although RORγt expression is markedly decreased in *Irf4^−/−^* T cells following treatment with IL-6 and TGFβ, retroviral overexpression of RORγt fails to fully restore IL-17 production during Th17 development, suggesting that IRF4 might be upstream of RORγt. IRF4-deficient T cells also express elevated Foxp3 levels under Th17 conditions, indicating that it might function to modulate RORγt/Foxp3 balance during iTreg to Th17 transition. It is unlikely that the impaired Th17 responses with reciprocal increase in numbers of Foxp3^+^ Tregs in IRF4-deficient animals is due its influence on STAT3 signaling as STAT3 signaling remains intact in absence of IRF4. A converse relation between IRF4 and FoxP3 is also reported, as Foxp3 knockdown results in marked decline in IRF4 expression in Treg cells (106). Moreover, IRF4-deficient Treg cells selectively fail to suppress Th2 response (106). IRF4-deficient Th17 cells also produce high levels of IFNγ in addition to elevated FoxP3 levels under Th17 conditions, suggesting that the TF plays an important role in plasticity of Th17 cells along both iTreg and Th1 axes (20).

Another TF, basic leucine zipper transcriptional factor ATF-like or BATF, a member of the AP-1 transcription family, is also essential for Th17 differentiation (18). Although BATF is expressed in Th1, Th2, and Th17 cells, BATF-deficient CD4 T cells display normal Th1 and Th2 differentiation but highly reduced IL-17 production under Th17 conditions. BATF also directly controls expression of the Bcl-6 and c-Maf TFs, both of which are needed for development of follicular helper T cells (107). After dimerizing with JunB, BATF regulates transcriptional activation of several Th17-specific genes by binding to promoters and intergenic regions of the *Il17a, Il17f, Il21*, and *Il22* genes. Similar to IL-6-deficient mice, BATF-deficient mice are resistant to EAE, but, unlike IL-6-deficient mice, BATF-deficient mice have normal frequencies of FoxP3^+^ CD4 T cells (18). However, despite comparable FoxP3 induction under iTreg condition, BATF-deficient T cells fail to downregulate Foxp3 in response to IL-6 and TGFβ. Similar to IRF4-deificency, retroviral overexpression of RORγt fails to restore IL-17 production in BATF-deficient Th17 cells, indicative of a potential synergistic interaction between RORγt and BATF. Nevertheless, unlike RORγt, absence of BATF prevents IL-6-mediated downregulation of FoxP3 despite intact STAT3 signaling, suggesting that BATF-mediated antagonism of iTreg programming can be a mechanism of promoting Th17 differentiation. Although overexpression of BATF in activated primary human T cells is known to decrease IL-2 production, neutralization of IL-2 failed to restore IL-17 production in BATF-deficient CD4 T cells (108). Additional studies are needed to understand how IL-2 signaling affects Th17 differentiation in absence of BATF *via* strengthening of counteractive FoxP3-dependent Treg programming during Th17 development. It is possible that during Th17 development, BATF restricts STAT5-mediated maintenance of FoxP3 expression *via* its suppressive effect on IL-2 induction, which results in downregulation of FoxP3, thereby preventing its inhibitory effect on RORγt. More recently, TCR signaling was found to promote assembly of IRF4/BATF complex on *Il17a/f* gene promoters, which makes the chromatin around this region more permissive to subsequent binding of lineage-specific TFs such as RORγt (23). As the assembly of IRF4/BATF is independent of Th17-polarizing cytokine-mediated signaling, these two TFs are designated as “pioneer” TFs, which promotes binding of subsequent lineage-specific cytokine-driven TFs to certain Th17-specific gene loci. Exactly how BATF and IRF4 crosstalk with STAT3 to govern Th17 differentiation is not clearly understood at present. As these TFs are induced in Th0 cells, it is tempting to hypothesize that they prime T cells to differentiate along different lineages contingent on the cytokine environment. Thus, while TGF-β- and IL-6-induced signals recruit STAT3/RORγt to a subset of BATF/IRF4 binding sites in *Il17a/f* promoters that are made accessible by their binding, Th1 or Th2 signals may recruit STAT1/T-bet or STAT6/GATA-3 to the promoter regions of the respective subset-specific genes. However, assigning a solitary function to these TFs would be undermining their complexity as their effects extend to other related subsets along the Th17 developmental axis.

### The Lineage-Associated Roles of Runx1, FoxP3 and T-bet

Runt-domain class of TFs (Runx) represents another family of proteins that broadly regulate CD4 Th cell differentiation (109). Of the three mammalian Runt domain TFs, Runx1 is required for normal hematopoiesis, including thymic T cell development. Runx1 influences Th17 differentiation by directly inducing *Rorc* expression as well as binding co-operatively with RORγt to facilitate *Il17* transcription (110). Runx1 also controls Treg function by co-operatively binding to FoxP3 to repress *Il2* transcription (111). FoxP3, by binding to Runx1, also inhibits RORγt–Runx1 complex-induced IL-17 expression. Therefore, a complex tripartite interaction among RORγt, Foxp3, and Runx1 has been proposed as a key regulatory mechanism governing balance between pro-inflammatory Th17 cells and anti-inflammatory Treg cells. In addition to the tripartite RORγt-Runx1–FoxP3 interaction, T-bet also binds to Runx1 to inhibit Th17 differentiation and prevent productive association of Runx1 with *Rorc* without directly repressing the *Rorc* promoter (112). Overexpression of Runx1 reverses this inhibitory effect of T-bet on IL-17A production by Th17 cells. Therefore, all three master TFs for iTreg, Th17, and Th1 subsets, namely, FoxP3, RORγt, and T-bet, physically interact with Runx1 to modulate transcriptional competence of *Il17* gene.

RORγt and Foxp3 are co-expressed in naive CD4 T cells exposed to TGFβ, and their co-expression is also found in a subset of intestinal CD4 T cells. During their development along iTreg–Th17 axis, Foxp3 inhibits the function of RORγt through an interaction involving the sequence encoded by exon 2 of FoxP3 (28). Pro-inflammatory cytokines such as IL-6, IL-21, and IL-23 relieve Foxp3-mediated inhibition of RORγt by suppressing FoxP3 expression in a STAT3-dependent manner. Therefore, plasticity of a developing CD4 T cell along the iTreg–Th17 axis is determined by the relative abundance of pro-inflammatory cytokines in the surrounding milieu followed by interactions between FoxP3, Runx1, and RORγt. Along the iTreg–Th17 axis of differentiation, T-bet is the only master TF that remains in a transcriptionally poised bivalent chromatin state, consisting of both permissive and non-permissive epigenetic marks, in differentiated iTreg as well as Th17 cells (113). Although several studies have indicated that Th17 responses are stronger in T-bet-deficient animals, T-bet is essential for generation of pathogenic Th17 cells (114–117). High T-bet induction is also necessary for differentiation and function of pathogenic TGFβ-independent Th17 cells (74). Surprisingly, suppression of T-bet also inhibits expansion of Th17 cells under specific circumstance *via* downregulation of IL-23R (118). Therefore, under certain circumstances, T-bet induction can well be necessary for differentiation of pathogenic Th17 cells and can serve a more intrinsic role in differentiation of pathogenic Th17 cells in addition to regulating late developmental plasticity of Th17 cells.

## Th17 Plasticity in Human IBD

**Figure 3:**
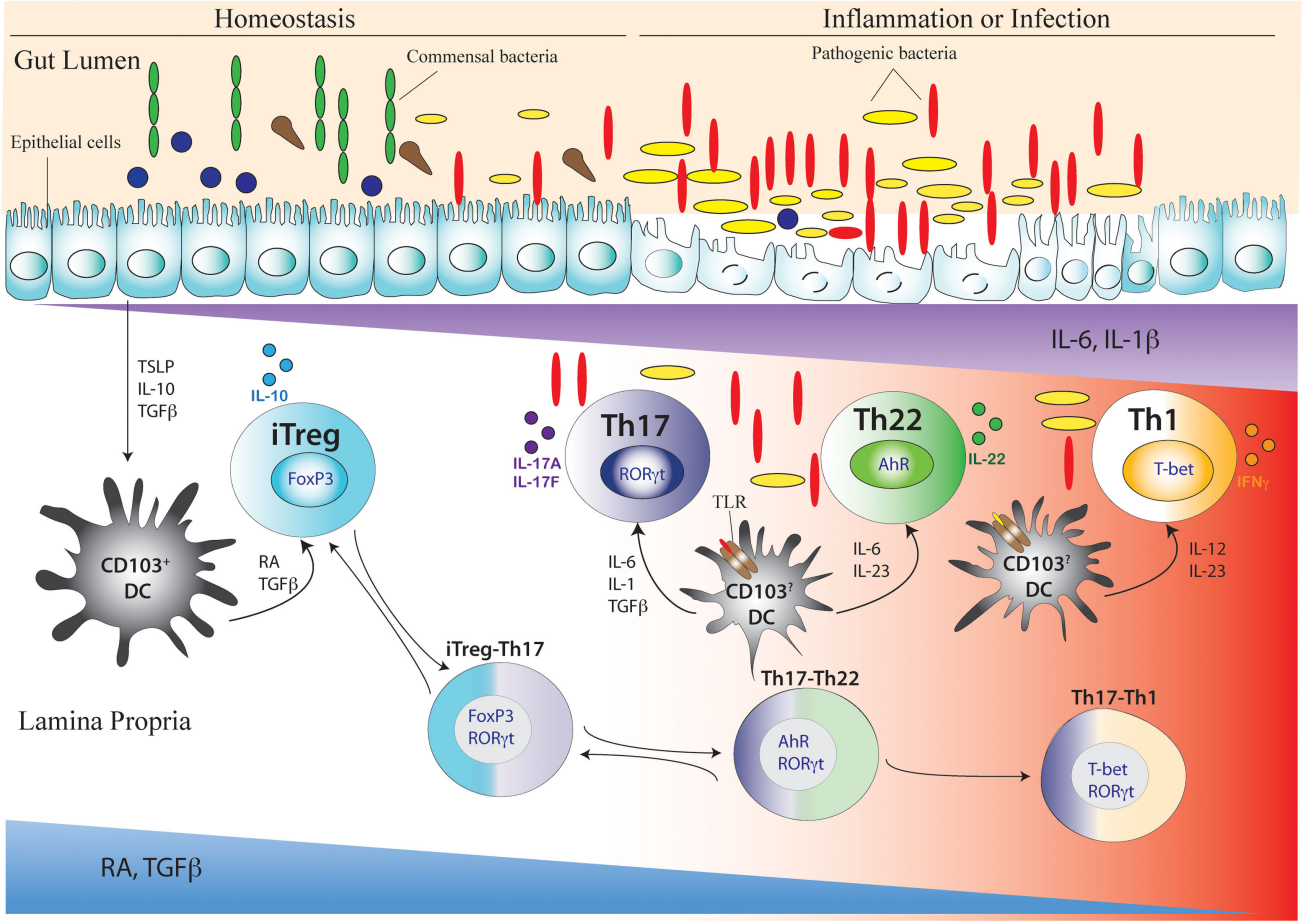
**Development of Th17 cell and its related intermediary cells during intestinal homeostasis and breakdown of homeostasis during inflammation or infection**. During intestinal homeostasis, CD103+ dendritic cells (DCs) of the intestine are conditioned by epithelial-cell-derived factors like thymic stromal lymphopoietin or TSLP, IL-10, and TGFβ. Conditioned DCs maintain tolerance by active secretion of retinoic acid (RA) that predominantly promote the differentiation of FoxP3-expressing inducible regulatory T (iTreg) cells. This process can also be facilitated by commensal microflora (green elongated and blue circular) like *Clostridum* sp. and *Bacteroides fragilis* that promote a tolerogenic phenotype in the DC by inducing secretion of TGFβ and other factors from intestinal epithelial cells. During intestinal homeostasis, iTreg cells are most abundant in the colonic lamina propria. Once intestinal homeostasis is broken by invasion of pathogenic microorganism (yellow elongated and red elongated) like *Citrobacter rodentium* or other bacterial species, the epithelial barrier is destroyed leading to loss or alteration of epithelial cell-derived immunosuppressive factors. As a result of which the tolerogenic state of DC is altered by activation of additional toll-like receptor (TLR) that induces DC to secrete proinflammatory cytokines like IL-6, IL-23, IL-1β, and IL-12. Accordingly, with decreasing spatio-temporal concentration of RA and TGFβ and increasing concentration of proinflammatory cytokines like IL-6, IL-23, and IL-1β, an infectious or inflammatory state allows induction of Th17, Th22, and Th1 cells. The induction of proinflammatory cytokines also induces the transition of iTreg cells to Th17 cells *via* intermediary FoxP3 and RORγt-coexpressing cells (iTreg–Th17). Reversion of Th17 cells to iTreg cells may also occur after resolution of inflammation. After maturation of Th17 cells, reduced TGFβ concentration may also lead to differentiation of Th22 cells via transitioning through AhR and RORγt co-expressing, intermediary Th17/Th22 cells that produce both IL-17 and IL-22 (Th17–Th22). Th22 cells can also transition to Th17 phenotype after being subjected to an environment supporting increased TGFβ concentration by shutting down IL-22 and initiating IL-17 production. During late Th17 development, chronic TCR stimulation of Th17 cells and/or the actions of IL-23 and IL-12 prompt the Th17 cells to transition into Th1 cells via T-bet and RORγt co-expressing Th17/Th1 cells that produce both IL-17 and IFNγ (Th17–Th1). Th1 cells are mostly fully committed, terminally differentiated cells that attain a “fixed” phenotype without any further fate alteration.

The immune system of the intestine is largest immune system in the body. The gut harbors billions of microbes, and RA-driven fine-tuning of specialized immune cells ensure proper intestinal homeostasis. The commensal intestinal microbiota affects the intestinal immune system in such way that homeostasis is achieved by enforcing equilibrium between Treg cells and Th17 cells, which are the two largest populations of CD4 T cell subsets present in the gut at homeostasis. However, this balance may be disrupted by aggravated immune assault on commensal microbe or invasion of pathogenic microbe leading to either an augmented pro-inflammatory environment or a diminished anti-inflammatory environment, resulting in pathological conditions of the intestine. Gut-resident CD103^+^ DCs, which favor tolerogenesis at homeostasis *via* their interaction with the local microenvironment rich in RA and TGFβ as well as with the intestinal epithelial cells, break down their tolerance on additional TLR activation by pathogenic microbes or by other pro inflammatory stimuli during inflammation. Inflammatory conditions also provoke induction of various pro-inflammatory cytokines from APCs that impacts fate conversion of CD4 T cells along the iTreg–Th17, Th17–Th22, and Th17–Th1 developmental axes (Figure 3). Accordingly, several intermediate phenotypes of CD4 T cells co-expressing FoxP3/IL-17, IL-17/IL-22, and IL-17/IFNγ are found in the intestine, mostly under inflammatory disease states.

Ulcerative colitis (UC) and CD are IBDs that are characterized by chronic inflammation of the intestine caused by an over-reactive host immune response against microbes or food antigens (119, 120). Although the precise etiology of IBD remains unclear, unrestrained activation of effector CD4 T cells has been regarded as a key factor in the pathogenesis of IBD. In chronic inflammatory diseases of the intestine, the balance of pro-inflammatory and regulatory cells plays a critical role in disease progression. Th17 cells are prevalent in IBD patients, who have increased IL-17A levels in their inflamed colonic mucosa (84, 121–123). Genome-wide association studies (GWAS) have indicated that IL-23R and at least five other Th17-associated genes are linked to susceptibility with CD (124–126). However, it is not clear how IL-23R polymorphisms predispose humans to CD by influencing Th17 differentiation.

Recent studies on developmental overlap among iTreg–Th17, Th22–Th17, and Th1–Th17 axes of cellular differentiation have compounded the problem of assigning specific functions of Th17 cells in the pathogenesis of IBD. At homeostasis, the intestine harbors an abundance of Treg cells. Patients with mutations in FoxP3 that result in non-functional or reduced numbers of Treg cells, suffer from severe intestinal inflammation (127). However, both CD and UC patients have increased numbers of FoxP3-expressing iTreg cells in their colonic lesions compared to non-inflamed areas, indicating that inflammation rarely results from a decrease in FoxP3-expressing iTreg population in human intestine (128). Although Treg cells are efficiently recruited to the inflamed colonic mucosa in IBD patients, it is possible that the function of iTreg cells might be compromised if they differentiate into Th17 cells as significantly higher prevalence of IL-17 and FoxP3 double-expressing CD4 T cells have been found in IBD patients (86). Compared to FoxP3-expressing cells alone, the IL-17 and FoxP3 co-producing population exhibit highly reduced suppressive ability. Alternatively, a prompt Th17 response arising from transitioning iTreg cells can also be beneficial under specific circumstances where Th17 cells have a protective role to play in eradicating pathogenic organisms and limiting inflammation. Studies from our group have shown that a failure in conversion of FoxP3-expressing iTreg cells into Th17 cells during enterobacterial infection in mice results in impaired bacterial clearance and enhancement of intestinal inflammation. Therefore, plasticity of Th17 cells along the iTreg axis can profoundly affect the disease outcome in patients where either failure of intermediate FoxP3^+^IL-17^+^ cells to gain effector Th17 function or their failure to suppress effector function can aggravate the inflammatory function in a context dependent manner.

The exact role of Th22 cells in the pathogenesis of CD and UC is unclear. After the developmental and functional characterization of Th22 cells, it was recognized that IL-22-producing CD4 T cells might comprise a population that is distinct from Th17 cells (8). Whereas Th17 cells promote recruitment of neutrophils and participate in tissue repair, Th22 cells promote mucosal healing through epithelial proliferation, restoration of epithelial barrier, and induction of antimicrobial molecules. GWAS in UC patients has identified a polymorphism located in chromosome 12,137 kb upstream of the *IL22* gene, suggesting a possible association of IL-22 expression with UC susceptibility (129). Although IL-22 is not detectable in the colonic mucosa of normal human subjects, IL-22 expression is readily detectable from CD4 T cells in the colonic mucosa of IBD patients. The frequency of IL-22-producing cells is increased in UC patients as well as CD patients, indicating a possible pro-inflammatory role in etiology of IBD (130, 131). However, a beneficial role for IL-22 has recently been suggested in human IBD (132). It was found that IL-17-producing Th17 cells alone are enriched in inflamed portions of the colon of IBD patients with a relative decline in Th22 cells that exclusively produce IL-22. Nevertheless, the decrease in Th22 cells observed during active inflammation is correlative and whether or not this is important for disease pathogenesis is currently unknown. Although, in acute intestinal inflammation Th22 cells are protective, careful studies need to be done to determine their function in chronic intestinal inflammation. It is also confounding that these two lineages developmentally overlap and one can arise out of the other along the Th17–Th22 axis: neutralization of TGFβ under Th17 condition completely suppresses IL-17 production and enhances IL-22 induction. During acute intestinal inflammation, IL-22-producing CD4 T cells emerge earlier than IL-17-producing CD4 T cells in the colonic lamina propria, indicating that Th17 cells might emerge as an offshoot of the Th22 differentiation pathway contingent on enhanced production of TGFβ (8). Although high levels of active TGFβ are produced in the inflamed tissues of IBD patients, it is not sufficient to stop mucosal inflammation (133). Indeed, in biopsies of UC patients where a selective depletion of Th22 was noted, TGFβ transcript expression was significantly higher compared to normal tissue of healthy controls, indicating that the TGFβ may play a role in preventing differentiation of Th22 cells in inflamed intestinal tissue of IBD patients (132).

The phenomenon of Th17 plasticity gives rise to another unique conundrum. During late development, Th17 cells can transition to IFNγ-producing Th1-like Th17 cells. CD, originally defined as a Th1-mediated pathology, has been recently reclassified as a Th17/Th1 phenomenon where mucosal tissue from patients has been found to produce IL-17 in addition of IFNγ (121, 134). Based on previous studies, it has been proposed that this ability of Th17 cells to transition into Th1 cells is a central mechanism that exacerbates immunopathology and that Th17/Th1 (ex-Th17) cells rather than Th17 cells alone play a critical role in IBD pathology (29, 30). In Th17-driven IBD, transition of Th17 precursors to Th1-like cells is absolutely required for disease, because Th17 cells deficient in IFN-γ or T-bet fail to induce intestinal inflammation (31). In CD patients, Th17 cells are found to produce both IL-17 and IFNγ, suggesting that Th17 plasticity contribute to disease pathogenesis (84). However, a therapeutic trial of administration of an anti-IL-17A monoclonal antibody to patients with moderate to severe CD had no therapeutic effect, and exacerbated the disease in some patients, suggesting a protective role of IL-17A in CD (135). It is tempting to speculate that Th17 cells and “Th1-like” Th17 cells might have disparate functions where “stable” Th17 cells are protective while “Th1-like” Th17 cells are pathogenic. In this scenario, immunological therapies aimed at depletion of IL-17-producing T cells might lead to adverse effect, as the beneficial Th17 cells will also be targeted. Therefore, unless the functions of these overlapping subsets are more clearly defined in IBD, generalized therapies might affect other developmental arms of Th17 cells due its inherent plasticity and overlap with related subsets of Th and regulatory T cells.

### The iTreg–Th17 Axis

The balance between factors promoting iTreg development and Th17 development is critical in determining homeostatic versus inflammatory condition in the intestine. As the early developmental programs of iTreg and Th17 cells are intimately linked, it is not surprising that intermediate phenotype along the iTreg–Th17 developmental axis exists *in vivo*. RORγt and FoxP3 co-expressing CD4 T cells are readily detected in the intestinal lamina propria (28, 136). Interestingly, in iTreg cells, differentiated in presence of TGFβ, both RORγt and FoxP3 are induced but FoxP3 *via* exon 2-encoded peptide inhibits RORγt activity through a protein–protein interaction (28, 136). During Th17 differentiation, action of pro-inflammatory cytokines such as IL-6 suppresses Foxp3 induction resulting in upregulation of RORγt expression. Although the competitive antagonism between FoxP3 and RORγt may negatively influence Th17 differentiation, expression of FoxP3 during early development stage may be integral part of Th17 differentiation as a substantial percentage of IL-17-producing CD4 T cells in the colon express FoxP3 at one time during their development (28). Purified FoxP3^+^ cells emerging during iTreg differentiation can differentiate into IL-17-producing cells when subjected to Th17 differentiation program (66). Similarly, human iTregs can differentiate into Th17 cells, particularly when exogenous IL-1β or IL-23 is present (137). RA, a vitamin A metabolite produced copiously by intestinal DC, favors FoxP3^+^ iTreg development by constraining Th17 development in an IL-2-dependent pathway (25, 26). RA signaling, mediated through intracellular retinoic acid receptors expressed in T cells, blocks the inhibitory effect of IL-6 on FoxP3 induction, thereby accentuating inhibitory effect of FoxP3 on RORγt (138). Additionally, RA can directly inhibit RORγt in CD4 T cells (139). RA can not only reciprocally modulate iTreg and Th17 differentiation but can also reverse the Th17 developmental program by converting it to FoxP3-expressing iTregs. Human myeloid-derived suppressor cells are able to convert fully differentiated Th17 T cells to FoxP3^+^ iTreg cells in a RA- and TGFβ-dependent pathway (140). Thus iTreg–Th17 axis of differentiation is more dynamic in nature than usually appreciated. In the process of investigating lineage association between iTreg and Th17 cells, study from our group has revealed a complex signaling network where IL-1 signaling crosstalks with tolerogenic RA signaling to modulate conversion along iTreg–Th17 developmental axis (26). A developing FoxP3-expressing iTreg cell, fortified with amplified pSTAT5 signaling by RA, has the ability to retain a dormant Th17 programming where IL-1 signaling-mediated amplified pSTAT3 expression becomes critical to shift the balance in favor of Th17 differentiation pathway during intestinal inflammation. This is primarily achieved *via* two newly discovered pathways—(a) direct enhancement of IL-2/STAT5 signaling in CD4 T cells by RA and (b) NF-κB-dependent SOCS3 repression by IL-1 resulting in sustained pSTAT3 signaling. Indeed, by favoring repressive pSTAT3 binding over pSTAT5 binding at an intronic enhancer of the *Foxp3* gene, IL-1-dependent potentiation of IL-6-driven STAT3 signaling directly subverts the Treg-stabilizing function of IL-2/STAT5 promoted by RA, thereby contributing to plasticity in the iTreg developmental program. This phenomenon explains why a substantial fraction of IL-17-producers in the intestinal lamina propria are found to express FoxP3 during their development. Pathogen-driven contraction of iTreg population with their concomitant conversion to Th17 cells in the gut lamina propria, might be effective to break tolerogenic environment of the gut, which otherwise would dampen the effector response required to thwart an invading microbe. Therefore, invoking conversion of the iTreg to Th17 effector cells would allow the fastest and most efficient generation of the intestinal effector response. Recently, a study has shown that Th17 cells can reversibly transdifferentiate into regulatory T cells during resolution of intestinal inflammation, which is contingent on AhR signaling (141). Therefore, fate commitment along the iTreg–Th17 developmental axis, besides depending on the interaction between lineage-specific TFs, is also governed by lineage-associated TFs. Besides fate inter-conversion between iTreg and Th17 lineages, late developmental axis of both subsets is also tied to Th1-like developmental program. Chromatin conformation of *Tbx21* locus remains in a transcriptionally poised state in both differentiated iTreg and Th17 cells. Accordingly, myelin-reactive IL-12-conditioned iTregs lose Foxp3 expression and express both T-bet and IFN-γ in a manner akin to Th17 to Th1 conversion (142). Therefore, plasticity along the iTreg–Th17 developmental axis also extends to Th1 subsets contingent on specific stimulatory conditions.

### The Th17–Th22 Axis

IL-22 belongs to the IL-10 family members of cytokine that is produced from innate immune cells as well as CD4 T cells and is assuming growing importance for its immunoregulatory functions in infection, inflammation, autoimmunity, and cancer (143). Like its sibling IL-10, divergent types of immune cells produce IL-22. A systematic study using cognate stimulation for different types of human immune cells-APC, NK, and T cells, indicated that CD4 T cells are the major producers of IL-22 (144). Initially IL-22 was identified as a Th1 cytokine in humans. Later on, Th17 cells were known to be the main producers of IL-22 that was co-expressed along with IL-17A and IL-17F (145, 146). However, the production of IL-22 by Th17 cells is somewhat counterintuitive, as TGFβ, which is critical for optimal Th17 differentiation, strongly inhibits IL-22 induction. However, a recent study demonstrated a TGFβ-independent pathway of Th17 differentiation that co-produced IL-17 and IL-22 along with high induction of T-bet, suggesting that a divergent lineage of Th17 cells can indeed produce IL-22 (74). Influence of T-bet on IL-22 induction was also reported by another study where retroviral transduction of T-bet in Th17 cells resulted in higher induction of IL-22 but downregulation of IL-17 (112). AhR and c-Maf are the two critical TFs that have been identified, which are responsible for induction of IL-22 from Th17 cells (22, 147). Nonetheless, later studies on human cells demonstrated existence of Th22 cells that exclusively produce IL-22 without IL-17 production (148–150). IL-23 was believed to be the principal cytokine required for IL-22 induction from Th17 cells despite the fact that IL-23 is dispensable for differentiation of Th17 cells (27). The conundrum was addressed in our study, which reported that IL-6, not IL-23, is the critical cytokine required for optimal differentiation of host-protective IL-22-producing Th22 subsets during *Citrobacter rodentium*-induced infectious colitis. It emerges that akin to shared requirement of TGFβ for iTreg and Th17 development, IL-6 is a common cytokine that is essential for driving both Th17 and Th22 differentiation. Moreover, as neutralizing TGFβ under Th17 conditions restores IL-22 induction, it is likely that Th17 and Th22 evolve from a common differentiation pathway. Curiously, emergence of IL-22-producing CD4 T cells in inflamed colonic lamina propria precedes development of IL-17-producing CD4 T cells during infectious colitis, indicating toward a possible biphasic development where an early “Th22” developmental phase is followed by late “Th17” phase contingent on local concentration of TGFβ. This aspect of early induction of IL-22 followed by late induction of IL-17 can also be ascribed to the differential activation of divergent TFs responsible for sequential induction of IL-22 and IL-17 induction from the same cell during its development. Comparative transcriptome analysis of Th22 and Th17 cells reveals differential expression of several hundred genes, among which are lineage-specific TFs such as T-bet and RORγt that are reciprocally regulated between these two subsets. This reciprocal regulation of *Tbx21* and *Rorc* expressions during Th22 and Th17 development is consistent with the role of TGFβ that suppresses T-bet and enhances RORγt. Despite the lower levels of AhR induction in Th22 cells compared to Th17, AhR contributes significantly to IL-22 expression in Th22 cells such that combined loss of T-bet and AhR actions results in complete abrogation of IL-22 expression. It is likely that AhR promotes transcriptional regulation of IL-22 in both Th17 and Th22 cells *via* binding to its cognate DRE present in *Il22* promoter. The observed interplay of AhR and T-bet in maximizing IL-22 production from Th22 cells also offers a possibility that IL-22 can also be produced by Th1 subsets in accordance to the original description of IL-22 as a product of human Th1 cells (144). As IL-23 induces both STAT3 and STAT4 in Th17 cells, the enhancing effect of IL-23 on IL-22 induction from both Th22 and Th17 cells might be partly explained by acquisition of IL-23-mediated Th1 competence in these cells. Accordingly, most of the IL-22-producing CD4 T cells of lamina propria co-express T-bet during infectious colitis (151). It is likely that IL-22-expressing Th17 cells are a distinct population compared to IL-22 expressing Th22 cells as inhibition of TGFβ signaling during infectious colitis specifically depletes IL-22-producing Th17 cells but increases the frequency of IL-22-producing Th22 cells (151). However, the functional specificities of IL-22-producing Th22 and Th17 cells remain unknown. In this regard, it is also important to note that Th22 cells express significantly lower levels of *Rorc* compared to Th17 cells suggestive of a supporting role of RORγt in IL-22 induction from Th22 cells. Despite validation of a protective role of IL-22-producing CD4 T cells in acute colitis, paradoxically it can also contribute to intestinal pathology during chronic colitis. IL-22-producing CD4 T cells are found to be pathogenic in a model of chronic colitis (152). Th22 cells are also found to be pro-inflammatory in the gastric mucosa of *Helicobacter pylori*-infected patients (153). However, a protective role of IL-22 has also been reported in IBD patients as reciprocal increase in Th17 and decrease in Th22 cell has been noted in inflamed colonic mucosa of patients due to increased levels of active TGFβ, which leads to loss of Th22 cells from the intestinal mucosa resulting in epithelial injury (132). Further studies are needed to understand plasticity and lineage relationship between Th17 and Th22 subsets in human IBD along with their individual roles in inflammation.

### The Th17–Th1 Axis

Although proximal signals governing Th1 development is clearly distinct from signals required for Th17 differentiation, committed Th17 cells can be induced to produce IFNγ by repeated TCR stimulation or on exposure to IL-12 (29). Th17 cells differentiated from naive CD4 T cells on re-stimulation yielded progeny that were a heterogeneous mix of cells expressing IL-17A, IL-17F, and IFNγ, either singly or in combination. However, re-stimulation in presence of exogenous TGFβ alone led to sustained expression of high IL-17A and IL-17F, while IL-23 failed to maintain IL-17A and IL-17F expression. Addition of exogenous IL-12 completely silenced IL-17A and IL-17F expression from Th17 cells, which rapidly transitioned into IFNγ-producing Th1-like cells in a STAT4 and T-bet dependent manner as the absence of the TFs prevented the transition. Later ChIP-Seq studies found out that plasticity of Th17 cells can be partly attributed to poised state of *Tbx21* locus in Th17 subset as permissive chromatin marks are retained in *Tbx21* locus of differentiated Th17 cells (113). It has also been confirmed that T-bet expression by Th17 cells is indeed required for colitis pathogenesis where IL-23 produced by innate cells of the intestine acts on developing Th17 cells to deviate their differentiation to Th1-like cells by upregulating T-bet, through a mechanism that is largely Stat4 dependent (31). Remarkably, *Gata3* locus also carries permissive chromatin marks in Th17 cells. Therefore, the obvious question to ask is why Th17 cells do not transition into Th2 cells? Indeed in a study it has been shown that *in vitro* differentiated Th17 cells can revert to both IFNγ-producing Th1 and IL-4-producing Th2 cells under Th1 and Th2 polarizing conditions (154). However, Th17 cells generated *in vivo* are often resistant to cross differentiation under Th1 and Th2 conditions (154). This suggests that the Th17 cells may have undergone pathways of differentiation *in vivo* that differ from those differentiated *in vitro*. Nevertheless, many human Th17 clones produce IFNγ and studies performed in humans clearly demonstrate the existence of Th1-like Th17 cells in both peripheral blood and gut tissues (84). Human Th17 and Th17/Th1 clones are similar in nature as they express IL-23R, RORγt, IL-12Rβ2 chain and T-bet. Similar to mouse Th17 cells, stimulation of human Th17 clones with IL-12 downregulated RORγt and upregulated T-bet and enabled the cells to produce IFNγ (84). In a fate-reporter mouse-based study, generated for tracking the fate of Th17 under inflammatory condition, almost all of the IFNγ-producing CD4 T cells in the spinal cord were found to be ex-Th17 cells (30). Th17 to Th1 conversion depends on IL-23 signaling that is required for the switch from IL-17A to IFNγ production during chronic inflammation. Transcriptional profile of Th17/Th1 cells drastically differs from conventional Th1 cells indicative of their dissimilar origin and function (155). On comparing the transcriptome signature of tyrosinase-related protein 1 (TRP-1)-specific Th1 and Th17 cells before and after adoptive transfer, transcriptome of these Th1/Th17 cells also differed markedly from classical Th1 cells despite induction of IFNγ and T-bet. Importantly, a population of transferred TRP-1-Th17 cells showed self-renewal capability and retained the capacity of sustained production of IL-17 without IFNγ co-production. This suggests that multipotency of Th17, capable of differentiating into Th1-like effector like progeny (Th1/Th17) as well as self-renewing IL-17-producing cells (Th17), are advantageous for host for tumor eradication. Based on the intrinsic association of T-bet with late Th17 developmental program, one cannot exclude the fact that T-bet might serve as an essential co-factor in development of pathogenic Th17 cells. Permissibility of *Tbx21* promoter in non-Th1 subsets indicates partial redundancy of T-bet in IFNγ transcription, as IFNγ locus remains non-permissive in Th17 cells. It has been suggested that T-bet plays a larger role than merely facilitating IFNγ transcription (156). Accordingly, T-bet-deficient mice are resistant to EAE whereas IFNγR^−/−^, IL-12Rβ2^−/−^, and IL-12p35^−/−^ mice, all of which lack critical components of the Th1-IFN-γ pathway, are highly susceptible to autoimmune diseases (157–161). Consistent with the notion of a wider unappreciated role of T-bet in Th17 differentiation, inhibition of T-bet suppresses the differentiation and/or expansion of myelin-specific Th17 cells and T-bet is found to be a critical factor for establishing the encephalitogenicity of Th17 cells (117). Therefore, T-bet induction might be necessary for plasticity of Th17 cells as well as the differentiation of pathogenic Th17 cells.

## Epigenetics of Th17 Differentiation and Its Relation to Plasticity

Epigenetic modifications denote heritable changes in gene expression by selective positioning of nucleosome that consists of two units of histones—H2A, H2B, H3, and H4—without any change in nucleotide sequence (162, 163). During Th differentiation, binding of lineage-specific TF to its cognate genes not only depends on its own induction but also on its accessibility to the cognate DNA binding elements, which is regulated by epigenetic modifications. Therefore, epigenetic changes modulate transcription of key genes linked to a specific Th lineage *via* alteration of their chromatin architecture, which can be permissive (transcriptionally accessible) or non-permissive (transcriptionally repressed). Following TCR ligation, major epigenetic changes are initiated by “pioneer” TFs and transduction of cytokine-driven STAT signaling. Lineage-specifying TFs subsequently act on the altered chromatin landscape brought about by STAT proteins and “pioneer” TFs to drive specific changes in gene expression. It is also being increasingly recognized that regulation of chromatin is a highly dynamic process that is kinetically altered during differentiation of T cell subsets.

One of the most studied epigenetic modifications on various Th lineages is methylation of specific histone proteins at specific lysine residues (72, 113, 164, 165). While histone trimethylation of H3K4 (H3K4me3) increases chromatic accessibility and transcriptional competence of a gene, trimethylation of histone H3K27 (H3K27me3) decreases chromatin accessibility and reduces transcriptional competence of a gene. Formation of DNase1 hypersensitivity sites in candidate loci of Th cells is another hallmark of transcriptionally active chromatin structure that can be mapped to functionally demarcate regulatory regions of genes where epigenetic changes have occurred (166). Besides methylation, histone acetylation is also critical for chromatin structure and associated with transcriptional competence of a gene. Acetylation of lysine 27 residue of histone H3 is a commonly studied epigenetic mark that is associated with active chromatin architecture. Several other forms of histone modifications affecting transcriptional competence also exist but have been less extensively studied in Th lineages. H3K4me3 and H3K27me3 epigenomes have been globally mapped in Th1, Th17, and iTreg susbets (113). Surprisingly, histone methylation patterns for lineage-specific TF genes exhibit both repressed and accessible epigenetic marks in unrelated subsets where they are not expressed. *Tbx21*, which encodes T-bet, a lineage-specific TF for Th1 subset, remains in a poised bivalent state in Th17 cells as well as iTreg cells, showing both repressive and permissive epigenetic modification in its locus. On the other hand, *Rorc* and *Foxp3* completely lacked permissive epigenetic modification in unrelated Th subsets such as Th1 and Th2 subsets. The bivalent epigenetic modification of *Tbx21* in Th17 and iTreg cells is consistent with the observed plasticity of these two subsets and their propensity to transition to Th1 subset. Besides remaining in a transcriptionally poised state during Th17 differentiation, T-bet can directly participate to alter permissive epigenetic marks in *Rorc* locus. T-bet binds directly at a specific region of *Rorc* locus in differentiated Th17 cells treated with IL-12 (72). IL-12 stimulation decreased permissive H3K4 methylation at *Rorc* locus, whereas TGFβ-stimulated cells retained H3K4 methylation—an effect that was reversed in the absence of T-bet. Therefore, epigenetic modifications influence Th17 differentiation at multiple levels encompassing the signature cytokine genes of Th17 subsets as well as TFs that regulate lineage plasticity. A global methylome analysis revealed that number of demethylated regions in Th17 cells far exceeds the number of demethylated regions present in Th1 cells (167). Th17 cells display an even greater number of demethylated regions compared to naïve T cells, suggesting that the high degree of demethylation might contribute to the plasticity of the Th17 cells.

Besides the master TFs, phosphorylated forms of STAT proteins also bind to their cognate gene locus and modulate epigenetic marks (168). pSTAT3 and pSTAT5 can bind to seven shared sites scattered in the promoters of the *Il17a* and *Il17f* genes as well as at distal locations relative to transcription initiation site (168). While binding of pSTAT3 correlated with permissive chromatin modifications associated with histone acetylation and H3K4me3 marks, pSTAT5 acted as a repressor of *Il17* transcription as its binding correlated with repressive chromatin modification. Of note, pSTAT5 binds directly to *Il12rb2* and *Tbx21* loci to facilitate Th1 differentiation (169). Therefore, how pSTAT5 binding can act both as activator and repressor of genes *via* alteration of epigenetic marks remains to be identified. BATF, another TF essential for Th17 differentiation, binds to conserved intergenic elements in the *Il17a/f* locus as well as directly to promoters of *Il17a, Il21*, and *Il22* genes (18). One of the BATF binding elements at the promoter region of *Il17a* gene directly overlaps with an identified *Rorc* binding element, suggesting that these two TFs interact by forming a stable ternary complex with DNA to modulate or stabilize transcriptional machinery of the *Il17a* gene. BATF, in conjunction with IRF4, also influences global histone acetyltransferase (p300) occupancy in Th17 cells, which is highly diminished in *Batf^−/−^* and *Irf4^−/−^* Th17 cells (23). However, RORγt deficiency had limited effects on p300 recruitment and H3K4 trimethylation, suggesting that it lacks a major role in epigenetic modification of its regulated genes. The co-operative binding of STAT proteins and the “pioneer” TFs followed by the binding of master TF not only determine the faithful transcriptional competence of their cognate loci but also ensure the maintenance of transcriptional competence through subsequent mitotic divisions. However, actions of other regulatory proteins during Th17 differentiation might disrupt the faithful maintenance of transcriptional competence through cell divisions by altering the epigenetic marks and contribute to Th17 plasticity.

## Th17 Plasticity: in Search of a Bona Fide Th17 Cells

If the original genetic makeup of a Th17 cell is irrevocably lost during an effector response due to its plasticity, how can there be an existence of a memory Th17 cell with its *bona fide* characteristics? How would the transitioned Th17 cells behave during a recall response? How is a Th17 program re-initiated upon re-encounter of a pathogen? In the context of these queries, one must distinguish between an infectious and autoimmune setting where Th17 cells play a role in disease pathogenesis. A study has shown that plasticity of Th17 cells along Th17–Th1 axis differed between inflammatory and autoimmune conditions (30). In contrast to the altered fate exhibited by Th17 cells during EAE where erstwhile Th17 cells cease IL-17A expression and gain IFNγ expression, acute fungal infection with *C. albicans* gives rise to Th17 cells that do not deviate to IFNγ-producers. The apparent difference in plasticity of Th17 cells under infectious and autoimmune conditions might be due to the longevity of the effector response. While microbes can be rapidly cleared from the system by a potent Th17 response that subsides with clearance, chronic stimulation of Th17 cells promotes an alternative fate. Accordingly, a single intranasal infection with *Streptococcus pyrogenes* gives rise to Th17 cells that exclusively produce IL-17 without IFNγ co-production, whereas repeated infection results in emergence of IL-17/IFNγ double producers (170, 171). Moreover, intranasal vaccination with *Klebsiella pneumoniae* leads to a dominant Th17 response with no change in IFNγ-producing population. Importantly, during a memory response, protection against *Klebsiella* was dependent on IL-17, but not on IFNγ, suggesting that protection was mediated by “committed” Th17 cells (171). This evidently shows, albeit indirectly, that a stable Th17 memory population can indeed be generated. Therefore, continued TCR activation of Th17 cells would have a bearing on the stability of the Th17 cells.

On comparing the fates of endogenously generated memory Th1 and Th17 subsets to bacterial infection, it has been found that Th1 cells induced by intravenous infection are more efficient at entering the memory pool than Th17 cells induced by intranasal infection (172). It was suggested that Th17-mediated immunity is short-lived because IL-17A-producing CD4 effector T cells do not survive to become memory cells. However, the possibility that some Th17 cells simply transitioned to Th1 and lost the capacity to produce IL-17A was not ruled out in this study. On the other hand, it has also been proposed that Th17 cells represent an “earlier,” less-differentiated, plastic and more stem cell-like state than Th1 cells. While investigating the anti-cancer responses mediated by Th17-polarized cells that effectively eradicated established tumors, it has been found that the acquisition of type 1 effector properties, including T-bet expression and the secretion of IFNγ *in vivo*, was required for the antitumor activity of Th17 cells (155). Whereas IFNγ-producing Th1 cells are terminal effectors prone to apoptosis that are short-lived and incapable of mounting a sustained antitumor effector response, Th17 cells are long-lived cells capable of maturational plasticity, which enables them to mount a sustained antitumor response.

Additionally, the initial priming cytokine environment during an inflammatory or autoimmune response can contribute to the fate determination of Th17 cells. This is demonstrated by the fact that *Candida*-specific Th17 differentiation, initiated in the presence of IL-12-producing fungal antigen-stimulated monocytes, produced significantly more IL-17/IFNγ co-producing cells than bacterial antigen-specific Th17 cells differentiated in presence of bacterial antigen-stimulated monocytes producing undetectable levels of IL-12 (32). Therefore, during the initiation of Th17 development in a given milieu, the relative abundance of Th17-promoting cytokines (IL-6, IL-1, IL-23, and IL-21) versus the Th17 inhibiting cytokines (IL-12, IL-27, IFN-γ, and IL-33) might well be instrumental in determining extent of plasticity of the developing Th17 cells. Contingent on their initial cytokine milieu, different populations of Th17 clones (stable or plastic) might emerge displaying intermediate stages of differentiation. Accordingly, a Th17 memory response can invoke proliferation of a stable IL-17-producing phenotype or a plastic IL-17 and IFNγ-co-producing phenotype. Hence to unravel the mystery of Th17 fate commitment with greater finesse, an all-inclusive approach needs to be taken to understand plasticity of Th17 cells where variables such as nature of APC, strength of TCR, and costimulation, physiologically relevant concentration of pro- and anti-Th17 cytokines are carefully considered.

## Conclusion and Perspectives

After its encounter with the priming microenvironment consisting of diverse APCs, a multitude of antigens and other microenvironmental factors, the journey of a native CD4 T cell toward becoming an antigen-specific Th17 cell can be broadly divided into three sequential terrains. During its journey through the first terrain, strength of TCR–pMHC interaction, strength of costimulation and other “non-cytokine”-induced factors co-operate with APC-generated cytokines (e.g., IL-6) to induce STAT proteins (e.g., STAT3, STAT1) and “pioneer” TFs such as BATF and IRF4. STAT proteins and pioneer TFs collaborate to initiate the lineage-specific developmental program by induction of STAT-responsive and IRF4/BATF-responsive genes, which include the lineage-specific master TF, RORγt. During its journey through the second terrain, STAT3-induced RORγt co-operates jointly with the “pioneer” TFs to alter chromatin accessibility of key Th17-related genes by epigenetic modification for making them transcriptionally permissive. In its final passage through the third terrain, an orchestration of complex networking of signaling events modulated by lineage-specific master TF, RORγt along with lineage-associated TFs (e.g., Runx1, AhR, and c-Maf), determines the stability of the Th17 developmental program through integration of various pro-inflammatory and anti-inflammatory environmental cues. Plasticity of Th17 cells is determined by the interplay of these variable factors acting across the three terrains.

The phenomenon of Th17 plasticity takes into account three distinct possibilities: (a) Th17 cells are inherently plastic in nature and are rarely terminally differentiated, (b) longevity of the Th17 effector response (“acute” versus “chronic”) determine development of “stable” versus “plastic” Th17 cells, and (c) initial priming environment of differentiating Th17 cells dictates Th17 plasticity. Taking into account all the three possibilities, the following unifying explanation can serve to formulate a likely model illustrating the journey of naïve CD4 T cell through the iTreg–Th17, Th17–Th22 and Th17–Th1 development axes. During iTreg differentiation, both FoxP3 and RORγt are induced by TGFβ alone where FoxP3 effectively suppresses RORγt *via* protein-protein interaction to prevent initiation of Th17 programming. Any pro-inflammatory stimulus imparted to these iTreg cells *via* STAT3-inducing cytokines such as IL-6 and IL-23 suppress FoxP3, which allows RORγt to become unrestricted and enables it to initiate Th17 programming in these cells. However, during Th17 development, T-bet still remains in a transcriptionally “quasi” permissive state that imparts an inherent plasticity in the Th17 cells. Nonetheless, overexpression of RORγt in Th17 cells can transcriptionally repress T-bet and effectively prevent its transition to Th1-like cells by an undefined mechanism (72). Therefore, due to antagonistic protein–protein interaction between FoxP3 and RORγt along the iTreg–Th17 axis, FoxP3 can potentially preclude the optimal levels of RORγt required to repress T-bet-mediated Th1 programming. With augmented suppression of FoxP3 by the pro-inflammatory cytokines, high RORγt-expressing Th17 cells can remain stable by repressing T-bet without transitioning to Th1-like cells. However, chronic TCR stimulation of dividing Th17 cells or their exposure to Th1-promoting cytokines such as IL-12 reprograms the *Tbx21* locus by altering its epigenetic marks making it increasingly more permissive. Once T-bet expression becomes co-dominant with RORγt, the developing Th17 cells growing in an environment of low TGFβ concentration, can either deviate into Th1-like subset co-producing IL-17 and IFNγ along the Th17–Th1 developmental axis or evolve into Th22 subset producing IL-22 along the Th17–Th22 developmental axis after additional integration of AhR-mediated signaling.

Therefore, retention of all the intermediate phenotypes arising during Th17 developmental program can be highly beneficial to an organism as it enables to maintain a dynamic balance between the regulatory and inflammatory axes. In a static mode of functionality, fully committed Th17 subsets will not have the flexibility to invoke an immune response that is specific to other subsets of Th cells. Given the fact that both IL-17/FoxP3, IL-17/IFNγ, IL-17/IL-22 can be detected *in vivo* under inflammatory condition, it is not difficult to envision that plasticity of Th17 cells can be tailored according to the need of the situation so as to increase immunological versatility and fitness. It will be exciting to see how the molecular events guiding the intricate developmental plasticity of Th17 cells reveal themselves in future.

## Author Contributions

RB conceptualized the review. SB and RB wrote a draft of the review. The figures were drawn by RB and SB. RB performed the final edits.

## Conflict of Interest Statement

The authors declare that the research was conducted in the absence of any commercial or financial relationships that could be construed as a potential conflict of interest.
